# Which Benefits and Harms of Using Fenugreek as a Galactogogue Need to Be Discussed during Clinical Consultations? A Delphi Study among Breastfeeding Women, Gynecologists, Pediatricians, Family Physicians, Lactation Consultants, and Pharmacists

**DOI:** 10.1155/2018/2418673

**Published:** 2018-04-23

**Authors:** Ramzi Shawahna, Sara Qiblawi, Haifa Ghanayem

**Affiliations:** ^1^Department of Physiology, Pharmacology and Toxicology, Faculty of Medicine and Health Sciences, An-Najah National University, Nablus, State of Palestine; ^2^An-Najah BioSciences Unit, Centre for Poisons Control, Chemical and Biological Analyses, An-Najah National University, Nablus, State of Palestine; ^3^Department of Medicine, Faculty of Medicine and Health Sciences, An-Najah National University, Nablus, State of Palestine

## Abstract

**Background:**

Breastfeeding women with hypogalactia are commonly recommended to use fenugreek as a galactogogue. This study aimed to achieve formal consensus among breastfeeding women and healthcare providers on which potential harms and benefits of using fenugreek need to be communicated and discussed during clinical consultations.

**Methods:**

A two-iterative round Delphi technique was used in two separate panels of breastfeeding women (*n* = 65) and healthcare providers (*n* = 56) to achieve formal consensus on a list of 24 and 16 items related to potential harms and benefits of fenugreek.

**Results:**

About 70% of the healthcare providers recommended quite often herbal remedies for breastfeeding women and about 68% of the women had been recommended to use herbal remedies many times by their healthcare providers. Consensus was achieved on 21 potential harms and 14 potential benefits of using fenugreek to enhance human milk supply that need to be discussed with breastfeeding women during consultations.

**Conclusion:**

Probably, potential harms and benefits of recommending fenugreek as herbal galactogogue for breastfeeding women seeking recommendations to increase their human milk supply need to be discussed during clinical consultations. Further observational studies are needed to assess what is being discussed in daily consultations when herbal remedies are recommended.

## 1. Introduction

Human milk has been recognized as the ideal form of enteral nutrition for term and preterm infants [1, 2]. Exclusive breastfeeding for the first six months of life has been shown to confer substantial benefits to both the mother and her infant [2]. Therefore, global health authorities recommend exclusive breastfeeding for all infants in the first six months of life which might then be continued alongside other solid foods as long as the mother and her infant desire [3]. According to recent estimates, only 37% of infants younger than six months of age are nourished exclusively on human milk in low and middle income countries [2]. In the US and Australia, about half of the infants were receiving human milk at all by 6 months and in the UK, only one-third were doing so [2]. These low figures cannot be explained merely by weak intentions to breastfeed because in the UK, more than 80% of women expressed their intention to breastfeed their infants [3, 4]. Certainly, some figures might be explained by insufficient human milk supply.

Insufficient breast milk supply was frequently reported as the main reason for discontinuing breastfeeding [5, 6]. Many women, particularly those who delivered preterm infants, suffer difficulties producing enough quantities of human milk. It is noteworthy mentioning here that even mothers of term infants under certain circumstances like maternal illness, cesarean delivery, excessive smoking, breast surgery, separation between mother and her infant, and psychosomatic illnesses might suffer insufficient human milk supply [3].

Nonpharmacological interventions remain the first line in managing insufficient human milk supply, although prescribing medications and recommending herbal galactogogues are common [7]. Women who discontinue breastfeeding as a result of insufficient human milk supply might be provided with adequate educational interventions regarding breastfeeding practices and/or might then be prescribed pharmacological agents to increase their human milk supply. Agents used to increase human milk supply are called galactogogues [5]. Metoclopramide and domperidone are the most commonly prescribed pharmacological galactogogues [5, 8, 9]. However, these agents have not received approval as safe and effective galactogogues from any health regulatory authority and currently are being used “off-label” [10, 11]. In addition, these agents are excreted in human milk and thus bear potential side effects and harms to infants [10–12]. Moreover, little guidance is available on the appropriate dosage of these agents when used as galactogogues [9, 13, 14]. Therefore, interventions to increase human milk supply using pharmacological agents might be complicated by safety concerns to women and their infants. Traditionally, herbal remedies have been viewed as good alternatives to prescription medications [15, 16].

In classical views, herbal remedies have been regarded as safe. Probably, this belief has emerged by advertising herbal remedies as mild, gentle, safe, and having unique attributes that are not found in prescription medications [15]. This myth was perpetuated by some healthcare providers when labeling herbal remedies as “natural” which are in turn mistakenly regarded as safe or in the worst case scenario, safer than prescription medications [17–19]. The myth that herbal remedies can never be harmful is perpetuated and commonly believed by many patients. However, this myth lacks scientific evidence. Herbal remedies contain chemicals that could resemble some active ingredients present in many prescription medications; thus, these chemicals would act by similar pharmacological mechanisms of action and have the ability to cause side effects and harm [15, 20]. It is noteworthy mentioning that herbal remedies are like prescription medications, have intended indications, are contraindicated in some cases, should be used with caution in some patients, and are associated with side effects [17, 18]. Therefore, herbal remedies should be recommended considering the 5 rights (right person, time, dose, frequency, and route of administration).

Herbal galactogogues have received considerable attention across different societies and cultures. Anecdotal reports of many herbal remedies supported their potential to enhance human milk supply. These herbal remedies include fenugreek, anise, caraway, fennel, milk thistle, and many others [16, 18, 21]. Fenugreek (*Trigonella arabica* Delile) which belongs to the pea family (Leguminosae) is the most widely used herbal galactogogue to enhance human milk supply in many countries [22]. Seeds of fenugreek, which is an annual herbaceous plant, are traditionally used as condiment and in folk medicine in many countries including the Indian subcontinent, China, and the Middle East [22]. A recent study in Kuwait showed that fenugreek was recommended for breastfeeding women with insufficient breast milk supply [23]. Anecdotal reports of the successful use of fenugreek as an herbal galactogogue have surfaced in 1940s. Little is known of the mechanism of action explaining how fenugreek might enhance milk supply. A theory suggested that fenugreek stimulate sweat production, and as the breast is a modified form of sweat gland, fenugreek might be able to stimulate the breast to supply an increasing amount of milk [21, 24]. There have been anecdotal reports of fenugreek increasing human milk supply in some 1200 breastfeeding women within 24–72 hours after consumption [24, 25]. Once the breast is stimulated, fenugreek consumption can be stopped as far the breast is stimulated and emptying continued. Fenugreek as galactogogue might be consumed in 2-3 capsules 3 times daily and each capsule might contain a variable quantity of fenugreek. At present, requirements for herbal products have not been standardized for consumption by patient [24]. It is noteworthy mentioning that the use of fenugreek is not free from side effects and has been associated with health related effects like excessive sweating, diarrhea, and worsening of asthma symptoms.

In modern healthcare delivery, patients are informed about the potential harms and benefits of therapeutic alternatives in order to develop their preferences. In general, making a decision on therapeutic alternatives involves balancing their potential benefits against their potential harms, taking into account the preferences of the patients. The benefits of informing patients are multifold, including better experienced quality of life, coping with side effects, and prevention of overestimation of the impact of therapy on cure [15]. Therefore, healthcare providers like gynecologists/obstetricians, pediatricians, lactation consultants, family physicians, and pharmacists who are often consulted by breastfeeding women seeking recommendations to enhance their human milk supply should discuss herbal galactogogues balancing their potential benefits again potential harms in case they wanted to opt for herbal remedies considering the preferences of the women concerned. Little was narrated on the potential harms and benefits of using fenugreek to enhance human milk supply in breastfeeding women that should be discussed during clinical consultations from the viewpoints of breastfeeding women, gynecologists/obstetricians, pediatricians, family physicians, lactation consultants, and pharmacists who are often consulted by breastfeeding women seeking recommendations to enhance their human milk supply. In general, recommendations on which potential harms and benefits of using fenugreek to communicate to and discuss with patients during clinical consultations are lacking. The aim of this study was to fill this gap in the literature.

The aims of this study was to achieve consensus among breastfeeding women, gynecologists/obstetricians, pediatricians, family physicians, lactation consultants, and pharmacists who are often consulted by breastfeeding women seeking recommendations to enhance their human milk supply on which potential harms and benefits of using fenugreek as a galactogogue that need to be communicated to and discussed with breastfeeding women during clinical consultations in which a decision to use fenugreek would be taken. This consensual core list of potential harms and benefits might promote congruence in daily healthcare delivery.

## 2. Materials and Methods

### 2.1. Gathering Information on Herbal Galactogogues Recommended in Clinical Practice

We contacted and interviewed 10 key contact healthcare providers who were often consulted by breastfeeding women seeking recommendations to use herbal galactogogues to enhance their human milk supply. We also interviewed 5 women who previously have sought recommendations and used herbal galactogogues to enhance their human milk supply.

The key contact healthcare providers were asked to provide their consent to include their initials and details as experts who were interviewed in this study. Participants were given the option to remain anonymous upon their desire. Key contacts provided their age, gender, academic degrees, specialty, number of years in practice, approximate number of breastfeeding women cared for on a monthly basis, herbal galactogogues they often recommend, the potential harms, and benefits of herbal galactogogues that need to be communicated to and discussed with breastfeeding women during the clinical consultations.

The key contact women were asked to provide their consent to include their initials and details as experts who were interviewed in this study. Women were also given the option to remain anonymous upon their desire. Women were asked to provide their age, academic degrees, employment status, and the potential harms and benefits of galactogogues that need to be communicated to and discussed with breastfeeding women during the clinical consultations. The detailed sociodemographic and practice details of the interviewees are provided as Supplementary Materials (Table S1).

Healthcare providers and women narrated their experience with herbal galactogogues in terms of benefits and harms. Herbal galactogogues mentioned by the interviewees are listed in Supplementary Materials (Table S2). All interviewees (healthcare providers and women) mentioned fenugreek as one of the most frequently recommended herbal galactogogues. As all interviewees mentioned fenugreek as a galactogogue, we decided to gather all potential harms and benefits of this herbal galactogogue that need to be communicated to and discussed with breastfeeding women during the clinical consultations between breastfeeding women and their caring healthcare providers in which fenugreek is to be recommended. All potential harms and benefits mentioned by the interviewees were collected. An extensive literature review was then conducted to gather other potential harms and benefits of using fenugreek that could be found in other studies [4, 6, 12, 13, 17, 18, 21, 22, 24–46]. All potential harms and benefits found in the previous studies were noted. Potential harms and benefits collected were rephrased into statements. We discarded all potential harms and benefits related to costs, convenience, and inconvenience. Statements were piloted for clarity and comprehensibility with 5 medical students and 5 lay persons. Some statements were revised based on the feedback of the pilot and all statements were compiled into a questionnaire.

### 2.2. The Consensual Technique

In this study, we used the Delphi technique as a tool to achieve formal consensus among panelists on which potential harms and benefits of using fenugreek by breastfeeding women to enhance their human milk supply should be communicated to and discussed with breastfeeding women during the clinical consultations between breastfeeding women and their caring healthcare providers. Recently, this formal consensus technique has evolved as one of the most frequently employed techniques in achieving consensus on issues lacking consensus in healthcare [15, 47–49]. This technique has many advantages over other techniques like round table meeting, focus, and nominal groups. The advantages of this technique include guarding the anonymity of the participants, ability to recruit panelists from different locations, convenience, saving the costs of bringing the panelists to a round table meeting, and immunity against individual domination of the discussion and influencing opinions of other panelists. The Delphi technique combines both quantitative as well as qualitative methods in which a multiround questionnaire system is completed in two or more iterative stages, known as rounds, over a period of time within one or more panels until consensus is achieved [50]. The panelists are often requested to express the level of their disagreement or agreement with a list containing items in a questionnaire. Consensus is defined a priori and items on which consensus was not reached in one round are included in a revised questionnaire for a subsequent round and the process is continued until reaching a conclusion that consensus on the remaining items is no longer likely to be achieved [15, 47–49]. Sharing statistical summaries and comments with the panelists in a trial to decrease the number of rounds needed to reach consensus on the items included is commonly practiced.

As the views and opinions of women and healthcare providers could be different from each other, we sought consensus in two separate panels [15]. A panel included healthcare providers who are often consulted by breastfeeding women seeking recommendations to increase their human milk supply and the other panel was composed of women who sought recommendations and used herbal galactogogues to enhance their human milk supply.

### 2.3. Panel of Healthcare Providers

A judgmental sampling technique was used to recruit panelists who were healthcare providers that were often consulted by breastfeeding women seeking recommendations to increase their human milk supply. Potential panelists were identified by personal contacts in the field. As breastfeeding women seeking recommendations to increase their human milk supply often consult gynecologists/obstetricians, lactation consultant nurses, pediatricians, family medicine specialists, and pharmacists, we aimed to recruit panelists with these specialties. Because the Delphi technique implies that the panelists have to be rich with experience and information to narrate, it is well-established that selection and recruitment of the panel members are among the most captious steps in the Delphi technique [15]. In the current study, panelists were approached and invited to participate as panel members based on their qualifications, specialty, and experience in the field of recommending herbal galactogogues for breastfeeding women seeking recommendations to enhance their human milk supply. Field researchers approached in person and invited the potential panelists to participate as panel members in the current study. Field researchers explained the design and objectives of the study to potential panelists and obtained their verbal consent before participation. The inclusion criteria were (1) having a basic or advanced qualification in a healthcare specialty related to being consulted by breastfeeding women seeking recommendations to enhance their human milk supply, (2) having a license to practice in Palestine, (3) having 5 or more years of practicing experience in a healthcare establishment attended by breastfeeding women seeking recommendations to enhance their human milk supply, and this was important as possessing previous knowledge of the subject being researched is a critical prerequisite for a panelist to take part in the Delphi technique [15], (4) consultation with 5 or more breastfeeding women on a monthly basis, (5) knowledge of the use of herbal galactogogues in enhancing human milk supply. In this study, 56 panelists were recruited and participated in the panel of healthcare providers. The panelists were not offered any financial incentives.

### 2.4. Panel of Women

In this study, snowball sampling was used to identify and recruit women who sought recommendations and used herbal galactogogues to enhance their milk supply. Potential panelists were identified using personal contacts in the field. Potential panelists were approached by field researchers in person and invited them to participate in this study. The field researchers explained the design and objectives of the study to the potential panelists and obtained their verbal consent before they were recruited to the panel. Women were invited and recruited to the panel when they met the inclusion criteria of (1) having breastfed at least one infant, (2) having been recommended at least once to use herbal galactogogues to enhance their human milk supply, (3) using one or more herbal galactogogue to enhance human milk production, and (4) willingness to take part in the current study. In this study, 65 women were recruited to the panel. Again, participants were not offered any financial incentives.

### 2.5. The Iterative Delphi Technique Rounds

#### 2.5.1. Delphi Round 01

In the first Delphi round, the questionnaire was given by hand to all 56 healthcare providers and 65 women. The questionnaire consisted of 2 sections. In the 1st section, the panelists were requested to disclose their sociodemographic details. The healthcare professionals provided their gender, age, academic qualifications, number of years in practice, specialty, how often they recommended herbal galactogogues for breastfeeding women in their clinical practice, and how often they communicated and discussed harms and benefits of herbal galactogogues that breastfeeding women might be consuming during clinical consultations. Female healthcare professionals were also requested to provide if they have breastfed before, and the number of infants they breastfed. Women were requested to provide their age, educational level, employment status, number of infants they breastfed, how often they have been recommended by their healthcare providers to use herbal remedies to enhance their human milk supply, and if they liked to have enough discussion with their healthcare providers on the potential harms and benefits of using herbal remedies during breastfeeding. The 2nd section of the questionnaire contained a list of 24 and 16 items related to potential harms and benefits, respectively, of using fenugreek as a herbal galactogogue to enhance human milk supply and the panelists were requested to express the degree to which they disagree or agree that each presented item needs to be communicated to and discussed with breastfeeding women during consultations on a Likert scale of 9 points [15, 47–49]. When the panelists scored 1–3, this indicated that they disagree with the importance of communicating and discussing the presented potential harm or benefit during the clinical consultation; that is, they are of the opinion that the presented potential harm or benefit should not be communicated to and discussed with breastfeeding women during the consultations. When the panelists scored 7–9, this indicated that they agree with the importance of communicating and discussing the presented potential harm or benefit to breastfeeding women during the clinical consultation; that is, they are of the opinion that the proposed potential harm or benefit should be communicated to and discussed with breastfeeding women during the consultation. When the panelists scored 4–6, this indicated that the panelists partially agreed with the importance of communicating and discussing the presented potential harm or benefit during the clinical consultation; that is, the panelists are inconclusive either the presented potential harm or benefit should be communicated to and discussed with breastfeeding women or not during the consultations. In this study, the panel members were requested and encouraged to add written comments to justify and/or qualify their scores on the Likert scale as in previous studies [15, 47–49].

#### 2.5.2. Definition of Consensus and Analysis of the Scores

Scores were analyzed using an Excel Sheet (Microsoft Excel 2013). The first quartile (Q1), median (Q2), third quartile (Q3), and the interquartile range (IQR) were computed for each item. Scores of both panels were analyzed separately. The data were analyzed using the same definitions of consensus used in previous studies [15, 47–49]. Briefly, the item included the list of important harms or benefits that need to be communicated to and discussed with breastfeeding women during the consultation when the median score fell between 7 and 9 and the interquartile range (IQR) fell between 1 and 2 and the item was excluded from the list of important harms or benefits that need to be communicated to and discussed with breastfeeding women during the consultation when the median score fell between 1 and 3 and the IQR fell between 1 and 2. However, the item was considered equivocal when the median score fell between 4 and 6 or the IQR was larger than 2. Equivocal items were included in a revised questionnaire for a subsequent Delphi round. In this study, consensus was based on at least 80% of the scores of the panelists in each panel separately.

#### 2.5.3. Delphi Round 02

A revised questionnaire containing all equivocal items was subjected to a second Delphi round. In a trial to reduce the number of Delphi rounds needed to reach consensus, we provided the panelists with (1) the median score and the IQR for each potential harm or benefit, (2) reminder of their own scores in the previous Delphi round, and (3) summary of the comments made by the panelists either to justify or qualify their scores.

Scores in this round were computed and analyzed according to the same definitions used in the previous Delphi round. After analyzing the scores and comments obtained in the second Delphi round, we came to a conclusion that it was unlikely that consensus would be achieved if we would conduct further Delphi rounds.

### 2.6. Ethical Considerations

This study received ethical approval from the Institutional Review Board (IRB) committee of An-Najah National University. We obtained verbal consent from all panelists before they participated in the current study. All views, opinions, and scores of the panelists weighed equally in the analysis.

## 3. Results

### 3.1. Response Rate

Questionnaires were completed by 56 healthcare providers who are often consulted by breastfeeding women and 65 women who breastfed before in the first Delphi round; therefore, the response rate was 100%. However, in the second Delphi round, 48 (85.7%) of the healthcare providers and 40 (61.5%) of the women completed and returned the questionnaire.

### 3.2. Characteristics of the Panelists Who Took Part in the Study

#### 3.2.1. The Panel of Healthcare Providers

In this study, the panelists who were healthcare providers were of different age groups, belonged to both genders, had variable number of years in practice, had different academic qualifications, and had various specialties. More than half of the panelists were male in gender, physicians, and 40 years, and older. About 56% of the panelists were either gynecologists/obstetricians, pediatricians, or family medicine specialists. About 59% of the panelists were in practice for 10 or more years. The detailed characteristics of the panelists are shown in Table 1.

#### 3.2.2. The Panel of Women

The women who took part as panelists in this study were of different age groups and had different educational levels and employment status. The majority of the women (about 85%) had a university degree and were 25 years and older. About 43% of the women breastfed 3 or more infants. The detailed variables of the women panelists who participated in this study are shown in Table 2.

### 3.3. Use of Fenugreek for Enhancing Human Milk Supply

About 70% of the healthcare provider panelists stated that they recommended quite often herbal remedies for breastfeeding women. About 68% of the women had been recommended many times by their healthcare providers to use herbal remedies for enhancing their human milk supply.

About 57% of the panelists discussed quite often herbal remedies that breastfeeding women could be using during their consultations with them. About 66% of the women stated that they would always like to have enough discussion with their healthcare providers on the potential harms and benefits of using herbal remedies for enhancing their human milk supply.

### 3.4. Potential Harms of Using Fenugreek to Enhance Human Milk Supply That Need to Be Communicated to and Discussed with Breastfeeding Women during the Clinical Consultation

In this study, consensus was achieved in both panels on 21 potential harms of using fenugreek to enhance human milk supply that need to be communicated to and discussed with breastfeeding women during the consultation. The detailed list of these items is shown in Table 3.

In general, there was consensus on 6 potential harms related to the anticoagulant effects of fenugreek, 3 potential harms related to the increased risk of abortion associated with using fenugreek, 4 potential harms related to comorbidities, 3 potential harms related to the effects of fenugreek on the blood pressure, 2 potential harms related to the effects of fenugreek on the blood glucose level, and 3 other potential harms related to the side effects of fenugreek.

### 3.5. Potential Benefits of Using Fenugreek to Enhance Human Milk Supply That Need to Be Communicated to and Discussed with Breastfeeding Women during the Consultation

In this study, consensus was achieved in both panels on 14 potential benefits of using fenugreek to enhance human milk supply that need to be communicated to and discussed with breastfeeding women during the consultation. A detailed list of these potential benefits is shown in Table 4.

In general, there was consensus on the potential benefits of fenugreek related to enhancing human milk supply and fertility. Consensus was also achieved to communicate and discuss other potential benefits of fenugreek related to its antioxidant, chemoprotective, immunomodulatory, antidepressant, and anti-infective properties with breastfeeding women.

### 3.6. Potential Harms and Benefits of Using Fenugreek to Enhance Human Milk Supply That Need or Need Not to Be Communicated to and Discussed with Breastfeeding Women during the Consultation Depending on the Individual Clinical Situation's Need

Consensus was not achieved on 3 potential harms and 2 potential benefits of using fenugreek to enhance human milk supply. These equivocal items are listed in Table 5. Whether to communicate and discuss these items during a clinical consultation was left to the choice of the healthcare provider and depending on the individual's needs.

## 4. Discussion

In the present study, we developed a consensual core list of important potential harms and benefits of using fenugreek as herbal galactogogue that should be communicated to and discussed with breastfeeding women seeking recommendations to increase their human milk supply from their caring healthcare providers in daily practice in two separate panels of women and healthcare providers. To the best of our knowledge, this consensual core list is the first attempt to develop guidance for healthcare providers to consult when recommending fenugreek-based herbal remedies to promote human milk supply in breastfeeding women seeking recommendations to enhance their human milk supply.

When gold standards are not existent, consensual techniques might provide alternative methods to reduce bias, enhance transparency, and validity of judgmental methods when developing certain criteria [15]. We believe that this consensual core list should appeal to healthcare providers and might be consulted to guide communicating and discussing potential harms and benefits of using fenugreek to promote human milk supply in breastfeeding women seeking recommendations to enhance their milk supply. Judgmental sampling was used to recruit panelists for the panel of healthcare providers and snowball sampling was used to recruit panelists for the panel of women. These nonprobability sampling techniques have long been regarded as biased [51]. However, for this study design and objectives, probability randomized sampling techniques were not feasible. Moreover, judgmental and snowball sampling techniques permitted the recruitment of panelists with prior knowledge of the subject being investigated who were rich in experience to narrate [15, 47–49]. The panel of healthcare providers was composed of gynecologists/obstetricians, pediatricians, family physicians, lactation consultants, and pharmacists. Those healthcare professionals would normally be consulted by breastfeeding women seeking recommendations to increase their human milk supply [17, 52]. Women who were recruited for the panel of women experienced inadequate human milk supply sought recommendations from healthcare providers and used herbal galactogogues.

The number of panelists in the panel of healthcare providers and panel of women was slightly larger than those used in previous studies in which consensus was sought on issues in healthcare [15, 47–49]. Currently, there is no consensus on the number of panelists in a panel of experts. Panel sizes varied greatly in previous studies and the sizes ranged from 10 over 1000 panel members [51].

In this study, a consensual core list of potential harms and benefits of using fenugreek as herbal galactogogue was developed to guide healthcare providers on what harms and benefits to discuss and/or address during the clinical consultation when opting to recommend fenugreek for breastfeeding women seeking recommendations to increase their human milk supply. Guidelines on what healthcare providers should communicate and discuss in terms of potential harms and benefits are currently lacking. We believe this consensual core list should help healthcare providers and change their behaviors during consultations with breastfeeding women seeking recommendations to increase their human milk supply. It has been argued that professionals would change behavior in response to recommendations they agree with rather than recommendations they do not agree with [15, 47–49].

The use of herbal remedies was reported to be high among women in Palestine [31, 53]. In this study, about 68% of the women reported that they were recommended to use herbal galactogogues many times. Similarly, about 70% of the healthcare providers reported that they recommend quiet often herbal galactogogues for breastfeeding women seeking recommendations to increase their human milk supply. Our findings were consistent with those previously reported by Bazzano et al. in the US, in which 70% of the healthcare providers surveyed indicated that they often recommend galactogogues [52]. Similarly, fenugreek was the most frequently recommended herbal galactogogue in Bazzano's study. In this study, about 68% of the women reported that they always wanted to have enough discussion with their caring healthcare providers on the potential harms and benefits of herbal remedies. Findings of this study were consistent with those reported in a previous study in which 76% of pregnant women stated that they would like to have enough discussion on the benefits and harms of ginger when recommended to alleviate symptoms of nausea and vomiting of pregnancy [15]. In this study, inclusion women who experienced human milk insufficiency and used herbal galactogogues in the panel of women ensured inclusion of the insecurities and concerns breastfeeding women would like their caring healthcare providers to address during clinical consultations. Interestingly, about 57% of the healthcare providers reported that they quite often address potential harms and benefits of herbal remedies during consultations with breastfeeding women.

In this study, the response rate was high in both Delphi rounds. This was consistent with other studies seeking consensus on issues in healthcare using the Delphi technique [15, 47–49]. This strength adds to the validity of the findings reported in this study. The panel of healthcare providers included panelists of both genders, different age groups, geographical locations, practice settings, specialties, and number of years in practice (Table 1). The panel of women included panelists from different geographical locations, age groups, number of breastfed infants, educational levels, and employment status (Table 2). This diversity adds to the strength and validity of the findings reported in this study.

In this study, consensus was achieved on potential harms related to the anticoagulant potential of fenugreek that need to be discussed and/or addressed during the clinical consultation (Table 3). These findings were consistent with those reported in another study in which consensus was achieved among healthcare professionals on addressing the potential harms and benefits of using ginger to manage nausea and vomiting of pregnancy, especially harms related to the anticoagulant potential of ginger [15]. Not surprisingly, patients were previously reported to want to hear more from their healthcare providers on the best ways to make out of the therapies they are taking [54, 55]. The anticoagulant effects of fenugreek were previously reported. A recent study showed that aqueous extract of fenugreek inhibited blood coagulation process* in vitro* and increased prothrombin time in a dose dependent manner in blood samples obtained from healthy individuals [41]. Drug-herb interaction between fenugreek and warfarin was also reported [26]. Professional groups like the American Society of Anesthesiologists have advised patients to stop consuming herbal therapies 2-3 weeks prior to surgery as a safety precaution to avoid risks of bleeding [15, 32]. Findings of this study suggested that both healthcare providers and women wanted the risks of bleeding associated with the use of fenugreek by breastfeeding women to communicate and discuss during the consultation in which fenugreek is recommended to be used. Informed breastfeeding women could be in a better position to decide whether to use fenugreek or opt for another safer alternative.

In this study, the panelists were of the opinion that the risks of abortion associated with using fenugreek should be communicated to and discussed with breastfeeding women during the consultations. Again, these findings were consistent with those reported in a previous study in which pregnant women and gynecologists agreed that the risks of abortion associated with using ginger for nausea and vomiting of pregnancy should be addressed during clinical consultations [15]. Previous studies showed that aqueous extract of fenugreek had potential teratogenic effects in humans and animals [33, 39]. Health regulatory bodies tend to recommend avoidance of herbal remedies even when the risks associated with their use are inconclusive. As a good example here, the German E Commission and the Finnish Food Safety Authority recommended that pregnant women should avoid ginger even though the risks of abortion associated with using ginger by pregnant women were largely inconclusive [56]. There could be cases in which breastfeeding women could become pregnant. The panelists in this study were of the opinion to warn women of these potential risks during the clinical consultations. Conservative views imply that women should be warned even when the potential risks are still inconclusive [38, 42].

The use of fenugreek could worsen the symptoms of some comorbidities. For example, fenugreek could worsen the symptoms of asthma [38, 42]. It has been recommended that individuals with chronic asthma and allergy should avoid consumption of fenugreek [28, 38]. Therefore, in this study, the panelists were of the opinion that this risk should be communicated to and discussed with breastfeeding women during consultations. Many breastfeeding women could be asthmatics and should be warned of this potential harm of using fenugreek. Again, breastfeeding women should be warned that fenugreek could cause nausea and vomiting which could be disturbing to the breastfeeding women and could have negative effects on their reported quality of life [39]. Fenugreek could be associated with diarrhea and excessive sweating for the breastfeeding women and their breastfed infants [34]. Severe diarrhea and excessive sweating could result in huge fluid loss that might lead to dehydration as well as serious consequences on the health of infants. These risks should be communicated to and discussed with breastfeeding during the consultations.

The findings of this study suggested that the risks associated with the effects of fenugreek on the blood pressure, blood glucose, and potassium levels should be communicated to and discussed with breastfeeding women during the consultations [29, 30, 37, 40]. Some breastfeeding women could be at risk of hypotension or hypoglycemia and should be warned against these risks when using fenugreek. The blood pressure and blood glucose levels of some breastfeeding women might be controlled by medications. Using fenugreek might have negative consequences of these controlled levels and hence, breastfeeding women at risk should be warned. Similarly, some breastfeeding women could be taking diuretics, laxatives, mineralocorticoids, or other hypokalemic agents. The panelists in this study were of the opinion that breastfeeding women should be warned that fenugreek might worsen their hypokalemia.

The panelists in this study agreed that benefits related to enhancing human milk supply should be communicated to and discussed with breastfeeding women during the consultations [22]. Enhancing human milk supply would be the primary anticipated effect of using fenugreek as a galactogogue. The panelists were of the opinion of informing the breastfeeding women recommended to use fenugreek of its antioxidant, estrogenic, and immunomodulatory properties [35, 43]. Chemoprotective effects against breast cancer and antidepressant effects of fenugreek might also be communicated to and discussed with breastfeeding women [27, 35, 43]. Many breastfeeding women might be concerned with breast cancer and postpartum depression and could be interested in learning about these potential benefits of fenugreek. Breastfeeding women might also be informed of the antibacterial, antifungal, and antipyretic effects of fenugreek [42]. Fenugreek might also be beneficial in controlling appetite, promoting weight loss, alleviate ulcer, and decreasing cholesterol and triglycerides levels. Many breastfeeding women could have gained weight during pregnancy and might be interested in decreasing their weight. Fenugreek might offer some help toward this end.

The opinions of the panelists were divisive on the importance of communicating and discussing potential effects of fenugreek in inducing thirst, marble like urine and sweat. Similarly, the opinions of the panelists were divisive whether to communicate to and discuss with breastfeeding women potential benefits of fenugreek related to enhancing cognition, memory, and its antiparkinsonian effects [36, 43]. These potential harms and benefits might be or might not be discussed depending on the needs of each individual case.

In general, care should be taken when breastfeeding women are recommended treatments as many medications and herbal remedies are excreted into the human milk. Therefore, both breastfeeding women and their breastfed infants could be vulnerable. In all cases, potential benefits should be weighed against potential risks considering other available safe alternatives. Similar measures should be applied when fenugreek-based herbal remedies are intended to be recommended as galactogogues for breastfeeding women seeking recommendations to enhance their human milk supply.

The findings of this study could be interpreted considering a number of limitations. First, this was an observational consensual study. Observing healthcare provider's recommendations of fenugreek in daily clinical practice and why it was recommended for breastfeeding women could have shown other findings. Second, in this study, we did not classify potential harms and benefits into major harms and minor harms. However, this classification goes beyond the scope and objectives of this study. Third, we did not hierarchize the potential harms and benefits in order of importance. The hierarchy would have helped healthcare providers to prioritize the information to be communicated and discussed in case they did not have enough time to go over all potential benefits and harms. Fourth, judgmental and snowball sampling techniques were used to recruit panelists for this study. These nonprobability sampling techniques are viewed as biased in conservative views. However, these techniques are commonly used for this type of studies as probability sampling techniques are not practically feasible. Finally, the number of panelists who participated in each panel was relatively small. However, there is no consensus on the number of panelists required for a Delphi technique. The number of panelists used in this study was slightly larger than sizes used in previous studies seeking consensus on issues in healthcare.

## 5. Conclusion

Panelists in this study were of the opinion that potential harms and benefits of recommending the use of fenugreek as herbal galactogogue for breastfeeding women seeking recommendations to increase their human milk supply need to be discussed during the clinical consultations. This could be important in promoting congruence in daily healthcare delivery, improving patient's experience with therapy, coping with side effects of the therapy, and enhancing patient reported quality of life. In this study, consensus was achieved on a core list of potential harms and benefits of using fenugreek as herbal galactogogue in breastfeeding women seeking recommendations to enhance their human milk supply that need to be communicated to and discussed with breastfeeding women during the consultations in which fenugreek-based herbal remedies are to be recommended. This consensual list might be consulted as guidance by healthcare providers who are often consulted by breastfeeding women seeking recommendations to enhance their human milk supply. Further randomized clinical trials are still required to establish evidence-based benefits and harms of fenugreek in breastfeeding women. More observational studies are needed to assess what is being communicated and discussed in daily consultations when herbal remedies are recommended.

## Figures and Tables

**Table 1:** Sociodemographic and practice details of the healthcare providers who are often consulted by breastfeeding women (*n* = 56).

Variable	*n*	%
Gender		
Male	30	53.6
Female	26	46.4
Age (years)		
<40	30	53.6
≥40	26	46.4
Have you breastfed before^a^		
Yes	19	73.1^b^
No	7	26.9^b^
Number of infants breastfed^a^		
0	7	26.9^b^
1	4	15.4^b^
2	3	11.5^b^
≥3	12	46.2^b^
Academic qualifications		
B.S.	21	37.5
M.S.	5	8.9
M.D.	28	50.0
Ph.D.	2	3.6
Specialty		
Gynecology/obstetrics	10	17.9
Pediatrics	5	8.9
Family medicine	16	28.6
Lactation consultant nurse	13	23.2
Pharmacist	12	21.4
Number of years in practice		
5–9	23	41.1
≥10	33	58.9
How often do you recommend herbal galactagogues for breastfeeding women?		
Quite often	39	69.6
Sometimes	17	30.4
How often do you discuss herbal galactagogues that breastfeeding women could be using during your consultations with them?		
Quite often	32	57.1
Sometimes	24	42.9

^a^The question was for healthcare providers who were female in gender. ^b^Percentages were based on the number of female panelists; B.S.: Bachelor of Science, M.S.: Master of Science, M.D.: Doctor of Medicine, and Ph.D.: Doctor of Philosophy.

**Table 2:** Sociodemographic details of the women who participated in this study (*n* = 65).

Variable	*n*	%
Age (years)		
<25	10	15.4
≥25	55	84.6
Educational level		
School	16	24.6
Bachelor's degree	37	56.9
Master's degree	12	18.5
Employment status		
Employed	39	60.0
Unemployed	26	40.0
Number of infants breastfed		
1	22	33.8
2	15	23.1
≥3	28	43.1
How often have you been recommended by your healthcare provider to use herbal remedies for enhancing your human milk supply?		
Many times	44	67.7
Once or a few times	21	32.3
Do you like to have enough discussion with your healthcare provider on the potential harms and benefits of using herbal remedies?		
Always	43	66.2
Sometimes	22	33.8

**Table 3:** Potential harms of using fenugreek to enhance human milk supply that need to be communicated to and discussed with breastfeeding women during the clinical consultation.

Item #	Potential harms	Round on which consensus was achieved	
		Panel of healthcare providers	Panel of women
	*Fenugreek has anticoagulant effects*		
1	Breastfeeding women who have a history of any clotting related disorder need to be warned not to take fenugreek	2	1
2	Breastfeeding women who have a history of vaginal bleeding disorder need to be warned not to take fenugreek	1	1
3	Breastfeeding women who are at risk of any bleeding disorder need to be warned not to take fenugreek	1	1
4	Breastfeeding women need to be warned that fenugreek might be associated with menstrual breakthrough bleeding	2	1
5	Breastfeeding women who are on anticoagulants need to be warned not to take fenugreek	2	1
6	Breastfeeding women who are on non-steroidal anti-inflammatory drugs (NSAIDs) need to be warned not to take fenugreek	2	1
	*Fenugreek might be associated with abortion*		
7	Women planning to become pregnant need to be warned that fenugreek is a potential utero-stimulant and might cause spontaneous abortion	2	2
8	Women with a history of previous miscarriage need to be warned not to take fenugreek	1	1
9	Women planning to become pregnant need to be warned that fenugreek might impair fetal development	1	1
	*Risks associated with using fenugreek on other co-morbidities*		
10	Breastfeeding women need to be warned that fenugreek might cause nausea and vomiting	2	2
11	Breastfeeding women need to be warned that fenugreek might cause diarrhea in the mother and her breastfed infant	2	1
12	Breastfeeding women with a history of asthma need to be warned that fenugreek might worsen the symptoms of their asthma	1	1
13	Breastfeeding women need to be warned that fenugreek might cause dehydration	1	1
	*Fenugreek could be associated with hypotension *		
14	Breastfeeding women with a history of or at risk of hypotension need to be warned not to take fenugreek	1	1
15	Breastfeeding women with a history of or at risk of dizziness need to be warned not to take fenugreek	2	1
16	Breastfeeding women who are on anti-hypertensive medications need to be warned not to take fenugreek	1	1
	*Fenugreek could be associated with hypoglycemia *		
17	Breastfeeding women with a history of or at risk of hypoglycemia need to be warned not to take fenugreek	2	1
18	Diabetic breastfeeding women whose disease is controlled by medications or insulin need to be warned not to take fenugreek	1	1
	*Other adverse effects*		
19	Breastfeeding women need to be warned that fenugreek might cause fever	2	1
20	Breastfeeding women need to be warned that fenugreek might cause excessive sweating	2	2
21	Breastfeeding women taking diuretics, laxatives, mineralocorticoids, and/or other hypokalemic agents need to be warned that fenugreek may worsen hypokalemia	2	1

**Table 4:** Potential benefits of using fenugreek to enhance human milk supply that need to be communicated to and discussed with breastfeeding women during the clinical consultation.

Item #	Potential benefits	Round on which consensus was achieved	
		Panel of healthcare providers	Panel of women
1	Breastfeeding women might be informed that fenugreek can be beneficial in enhancing their human milk production	1	1
2	Breastfeeding women might be informed that fenugreek might improve their fertility	2	2
3	Breastfeeding women might be informed that fenugreek has antioxidant properties	2	2
4	Breastfeeding women might be informed that fenugreek has estrogenic effects	2	1
5	Breastfeeding women might be informed that fenugreek has immunomodulatory effect	1	1
6	Breastfeeding women might be informed that fenugreek has chemo-protective effect against breast cancer	1	1
7	Breastfeeding women might be informed that fenugreek may decrease plasma cholesterol and triglycerides levels	1	1
8	Breastfeeding women might be informed that fenugreek may have antidepressant activity	2	1
9	Breastfeeding women might be informed that fenugreek may have antibacterial activity	1	1
10	Breastfeeding women might be informed that fenugreek may have antifungal activity	1	1
11	Breastfeeding women might be informed that fenugreek could decrease their appetite, especially those with a history of eating disorders	2	1
12	Breastfeeding women might be informed that fenugreek can enhance weight loss	2	1
13	Breastfeeding women might be informed that fenugreek might have antipyretic activity	2	1
14	Breastfeeding women might be informed that fenugreek may alleviate symptoms of ulcer	2	1

**Table 5:** Potential harms and benefits of using fenugreek to enhance human milk supply that need or need not to be communicated to and discussed with breastfeeding women during the consultation depending on the individual clinical situation's need.

Item #		Panel of healthcare providers				Panel of women			
		Round 1		Round 2		Round 1		Round 2	
		M	IQR	M	IQR	M	IQR	M	IQR
	*Potential harms *								
1	Breastfeeding women need to be warned that fenugreek may induce thirst	5	2	5	3	6	2	5	3
2	Breastfeeding women need to be warned that fenugreek may be associated with maple syrup like urine	4	3	5	2	5	2	6	3
3	Breastfeeding women need to be warned that fenugreek may be associated with maple syrup like sweat	5	2	4	3	4	3	4	2
	*Potential benefits*								
1	Breastfeeding women might be informed that fenugreek may have antiparkinsonian activity	4	4	5	3	6	2	6	3
2	Breastfeeding women might be informed that fenugreek may improve memory and cognition	4	2	4	3	5	3	5	3

M: median, IQR: interquartile range.

## Supplementary Materials

Supplementary Materials Supplementary Table S1 provides the sociodemographic and practice details of the key contacts who were interviewed in this study (*n* = 15). Supplementary Table S2 provides details of the plants cited by the key contacts who were interviewed in this study (*n* = 15).
