# Supplementary material for: Which Benefits and Harms of Using Fenugreek as a Galactogogue Need to Be Discussed during Clinical Consultations? A Delphi Study among Breastfeeding Women, Gynecologists, Pediatricians, Family Physicians, Lactation Consultants, and Pharmacists
Source: Evid Based Complement Alternat Med. 2018 Apr 23;2018:2418673. doi: 10.1155/2018/2418673 (PMC5937604; doi:10.1155/2018/2418673)
Supplement: Supplementary Materials — Supplementary Table S1 provides the sociodemographic and practice details of the key contacts who were interviewed in this study (n = 15). Supplementary Table S2 provides details of the plants cited by the key contacts who were interviewed in this study (n = 15). [file 2418673.f1.docx]

**Table S1: Sociodemographic and practice details of the key contacts who were interviewed in this study (*n = 15*)**

| **Healthcare providers^*^** | | | **Women^**^** | | |
| --- | --- | --- | --- | --- | --- |
| **Variable** | **n** | **%** | **Variable** | **n** | **%** |
| **Age (years)** |  |  | **Age (years)** |  |  |
| 30-39 | 3 | 30.0 | < 40 | 2 | 40.0 |
| 40-49 | 3 | 30.0 | ≥ 40 | 3 | 60.0 |
| 50-59 | 4 | 40.0 | **Educational level** |  |  |
| **Gender** |  |  | School | 2 | 40.0 |
| Male | 3 | 30.0 | University | 3 | 60.0 |
| Female | 7 | 70.0 | **Employment status** |  |  |
| **Academic degree** |  |  | Employed | 2 | 40.0 |
| BSc | 3 | 30.0 | Unemployed | 3 | 60.0 |
| MSc | 2 | 20.0 |  |  |  |
| MD | 5 | 50.0 |  |  |  |
| **Specialty** |  | 0.0 |  |  |  |
| Gynecology/obstetrics | 4 | 40.0 |  |  |  |
| Pediatrics | 1 | 10.0 |  |  |  |
| Lactation consultant | 5 | 50.0 |  |  |  |
| **Number of years in practice** | |  |  |  |  |
| < 10 | 3 | 30.0 |  |  |  |
| ≥ 10 | 7 | 70.0 |  |  |  |
| **Approximate number of breastfeeding women cared for on monthly basis** | | |  |  |  |
| < 30 | 4 | 40.0 |  |  |  |
| ≥ 30 | 6 | 60.0 |  |  |  |

^*^ Initials of the healthcare providers: NK, IB, FK, HA, FK, MS, HM an three wished to remain anonymous

^**^ Initials of the women: SM, MT, KM, SM, AI

BSc: Bachelor of Science, MSc: Master of Science, MD: Doctor of Medicine

**Table S2: Plants cited by the key contacts who were interviewed in this study (*n = 15*)**

| **#** | **The English and (Latin) name of the plant** | **Family** | **Number of times cited** |
| --- | --- | --- | --- |
| **1** | Fenugreek (*Trigonella arabica* Delile) | Leguminosae | 15 |
| **2** | Anise (*Pimpinella anisum* L.) | Apiaceae | 11 |
| **3** | Milk thistle (*Silybum marianum* (L.) Gaertn.) | Compositae | 11 |
| **4** | Fennel (*Foeniculum vulgare* Mill.) | Apiaceae | 11 |
| **5** | Nigella (*Nigella arvensis* L.) | Ranunculaceae | 10 |
| **6** | Sesame (*Sesamum indicum* L.) | Pedaliaceae | 10 |
| **7** | Caraway (*Carum carvi* L.) | Apiaceae | 9 |
| **8** | Roselle (*Hibiscus sabdariffa* L.) | Malvaceae | 8 |
| **9** | Lentil (*Lens culinaris* Medik.) | Leguminosae | 7 |
| **10** | Cinnamon (*Cinnamomum verum* J.Presl) | Lauraceae | 7 |
| **11** | Palm dates (*Phoenix dactylifera* L.) | Arecaceae | 6 |
| **12** | Moringa (*Moringa oleifera* Lam.) | Moringaceae | 4 |
